# The Impact of *Streptomyces griseus* Protease Reserved for Protein Evaluation of Ruminant Feed on Carbohydrase Activity during Co-Incubation

**DOI:** 10.3390/ani14131931

**Published:** 2024-06-29

**Authors:** Paul Okon, Sandra Liebscher, Andreas Hans Simon, Monika Wensch-Dorendorf, Martin Bachmann, Frank Bordusa, Annette Zeyner

**Affiliations:** 1 Institute of Agricultural and Nutritional Sciences, Martin Luther University Halle-Wittenberg, 06120 Halle (Saale), Germany; 2 Charles Tanford Protein Centre, Institute of Biochemistry/Biotechnology, Martin Luther University Halle-Wittenberg, 06120 Halle (Saale), Germany

**Keywords:** *Streptomyces griseus* protease test, ruminal protein degradation, co-incubation, in vitro

## Abstract

**Simple Summary:**

Ruminal feed protein degradation can be estimated in vitro using a protease. Plant proteins associated with carbohydrates might, however, not be completely hydrolyzed by the protease alone. For this reason, carbohydrase is additionally required in the test. The co-incubation of carbohydrase and protease appears to be a suitable methodological approach. It is not clear, however, whether this approach would be feasible as the carbohydrase could be inhibited by the protease. The objective of the present study was thus to investigate the effect of co-incubation of carbohydrase (α-amylase, a cell wall-degrading enzyme mixture) and protease on the hydrolysis of carbohydrates. The co-incubation of protease tended to reduce the hydrolysis of carbohydrates by carbohydrase.

**Abstract:**

For protein evaluation of feedstuffs for ruminants, the *Streptomyces griseus* protease test provides a solely enzymatic method for estimating ruminal protein degradation. Since plant proteins are often structured in carbohydrate complexes, the use of carbohydrase during the test might improve its accuracy. It is advisable to co-incubate protease and carbohydrase, risking that the carbohydrase activity is reduced under the influence of the protease. The present study was conducted to investigate this impact by using α-amylase or the multi-enzyme complex Viscozym^®^ L as carbohydrase. The detection of active protease was determined fluorescence photometrically using internally quenched fluorogenic substrates (IQFS). Cellulose, pectin, and starch degradation were determined spectrophotometrically using 3,5-dinitro salicylic acid as a colorimetric agent. The *Streptomyces griseus* protease mixture proved to be active for the selected IQFS immediately after the start of measurements (*p* < 0.05). Starch hydrolysis catalyzed by α-amylase or Viscozym^®^ L, respectively, was decreased by co-incubation with protease mixture by maximal 3% or 37%, respectively, at 5 h incubation time (*p* > 0.05). Pectin and cellulose hydrolysis catalyzed by Viscozym^®^ L, respectively, was not significantly influenced by co-incubation with a protease mixture (*p* > 0.05). Although a decrease in carbohydrase activity during co-incubation with *Streptomyces griseus* protease occurred, it was only numerical and might be counteracted by an adapted carbohydrase activity.

## 1. Introduction

Ruminal protein degradation is an important parameter for assessing the protein quality of feed for ruminants. Various approaches can be used to determine ruminal feed protein degradation. Alternative methods not based on the use of animals appear to be valuable due to their standardized and routine application. An example of an alternative method for determining ruminal protein degradation is the standardized *Streptomyces griseus* protease test. The method is based on the incubation of a feedstuff at different time points with the *S. griseus* protease mixture consisting of endo- and exopeptidases [1]. The advantage of the *S. griseus* protease mixture lies in its diverse enzymatic composition, which ensures extensive protein degradation [2]. The *S. griseus* protease test was used at pH 6.75 and 39 °C on a broad spectrum of feedstuffs in comparison to in situ degradation data. However, the accuracy of the *S. griseus* protease test is limited by feed-specific complexes consisting of proteins and carbohydrates such as fiber and starch [3]. Such complexes cannot be hydrolyzed by proteases [4,5,6], which is why additional carbohydrases with fiber and/or starch-hydrolyzing activities are probably necessary in *S. griseus* protease test. The commercial enzyme preparations Viscozym^®^ L and Termamyl^®^ 2X (α-amylase) were used in pre-incubation with a supporting effect on feed protein degradation by *S. griseus* protease [7]. Viscozym^®^ L contains several cell wall-degrading enzymes such as cellulases, hemicellulases, pectinase and ß-glucanase and degrades starch and cellulose to measurable sugar monomers [8].

Methodologically speaking, carbohydrases and proteases could be incubated either separately in two successive steps as pre-incubation [4,5,7] or together as co-incubation [9]. However, the pre-incubation process requires additional incubation steps, including centrifugation and pH adjustment, for optimal enzymatic activity [4,5]. The co-incubation offers the potential to reduce effort and time and improve method handling. However, contradictory results have been reported regarding the co-incubation of proteases and carbohydrases, and it appears possible that added carbohydrases can be degraded or inactivated [10,11]. 

The aim of the study was to investigate the effects of the co-incubation of the *S. griseus* protease and carbohydrase (α-amylase/Viscozym^®^ L) on the release of reducing sugars from different carbohydrates at different incubation times. If the co-incubation of carbohydrase and *S. griseus* protease appears to be possible, the addition of carbohydrase might be a suitable approach to increase the accuracy of the *S. griseus* protease test by reducing the protein-carbohydrate complexes. 

We hypothesized that during co-incubation, the *S. griseus* protease mixture affects the carbohydrase Viscozym^®^ L and α-amylase in the hydrolysis of carbohydrates by reducing the conversion.

## 2. Materials and Methods

The study was divided into a preliminary and a main experiment. The preliminary experiment was conducted:

1. to determine whether the *S. griseus* protease mixture is active at 39 °C and pH 6.75, measuring the hydrolysis of two peptides through an increased fluorescence signal (fluorescence assay) and,
2. to investigate the relationship between increasing doses of *S. griseus* protease mixture on the release of reducing sugars due to the degradation of starch by α-amylase (absorbance assay).

The main experiment aimed to investigate the effect of *S. griseus* protease on different carbohydrates during co-incubation with carbohydrase, based on the results of the preliminary experiment. In general, Viscozym^®^ L and *Streptomyces griseus* protease are both mixtures of enzymes, making it difficult to detect activity with just one substrate. Therefore, different substrates are incubated with the respective enzyme.

### 2.1. Enzymes and Buffer Solution

The following enzyme preparations were used after the first opening: Viscozym^®^ L (V2010, Merck KGaA, Darmstadt, Germany) and α-amylase (Termamyl^®^ 2X, Univar Solutions, Essen, Germany). According to the manufacturer, Viscozym^®^ L contains several cell wall-degrading enzymes such as arabanase, cellulase, hemicellulase, pectinase and ß-glucanase. The manufacturer specifies the activity of Viscozym^®^ L as ≥100 fungal ß-glucanase units per g at a concentration of 1.10–1.30 g/mL. One fungal ß-glucanase unit is the enzyme required to hydrolyze barley β-glucan to reducing carbohydrates at standard conditions (pH 5.0, 30 °C, 30 min reaction time) at 1 µmol glucose per min [12].

The activity of α-amylase is given as 240 kilo novo units alpha-amylase with a concentration of 1.25 g/mL. One kilo novo unit is the amount of enzyme that hydrolyzes 4870 mg of starch per hour under standard conditions (pH 5.6, 37 °C and 0.3 mM Ca^2+^) [13]. 

The *S. griseus* protease stock solution contained 0.58 U/mL [14] of nonspecific proteases (Merck KGaA, Darmstadt, Germany). This concentration was used for all reactions. One unit is defined as the amount of enzyme that hydrolyzes casein, producing a color equivalent by the Folin–Ciocalteu reagent to 1.0 μmol (181 μg) of tyrosine per min at pH 7.5 and 37 °C.

The buffer solution was prepared as a borate-phosphate buffer (23 mM NaH_2_PO_4_ and 88 mM Na_2_B_4_O_7_ with pH 6.75) [1]. The borate-phosphate buffer was used in all experiments.

### 2.2. Substrates

For protease activity test, two internally quenched fluorogenic substrates (IQFS) peptides [15] of the general sequence Abz-Ala-Ala-Xaa-Phe-Ala-Ala-Lys-(Dnp) (Abz: 2-amino benzoic acid; Ala: alanine; Dnp: 2,4-dinitrophenol; Phe: phenylalanine; Lys: lysine; IQFS 1: Xaa = Alanine; IQFS 2: Xaa = Arginine) were used. The N-terminal 2-Abz functionality serves as a fluorescence donor. 2,4-dinitrophenol located at the N-terminal end of the lysine peptide acts as a fluorescence acceptor, resulting in a quenched fluorescence signal of the intact substrate. Any proteolytic event within the peptide goes along with a respective fluorescence signal (wavelength (λ) of fluorescence excitation (λex) = 320 nm, wavelength (λ) of fluorescence emission (λem) = 420 nm), which was measured in a fluorescence plate reader (Greiner Bio-One GmbH, Frickenhausen, Germany). Peptides were built up manually onto 2-chlorotrytil resin via standard fluorenylmethoxycarbonyl chemistry [16,17].

The following polysaccharides were used as substrates for carbohydrase: cellulose (R200 Vitacel J. Rettenmaier & Söhne GmbH + Co KG, Rosenberg, Germany), starch (CAS-No: 9005-25-8, Merck KGaA, Darmstadt, Germany) and pectin (agroPect Instant; agro Food Solution GmbH, Werder (Havel), Germany). Pectin was dissolved under continuous stirring with a magnet stirrer, cellulose and starch each through manually shaking in a borate-phosphate buffer. Cellulose, pectin and starch were dissolved to obtain a concentration of 20 mg/mL (*w*/*v*) [18]. Pectin was completely entirely dissolved, and cellulose and starch formed an emulsion in the buffer solution. All substrates were freshly prepared. 

### 2.3. Fluorescence Assay

Fluorescence measurements were conducted in a 96-well flat-bottom fluorescence plate (Greiner Bio-One GmbH, Frickenhausen, Germany) at 39 °C. IQFS peptides were dissolved in borate-phosphate buffer (pH 6.75) in a concentration of 100 µM in the presence and absence of pectin (20 mg/mL *w*/*v*) [18], respectively. A total of 100 µL of respective IQFS solution were pipetted into the 96-well microplate and 2.5 µL *S. griseus* protease solution were added. The increasing fluorescence measurement started immediately after the protease solution was added using a NOVOstar microplate reader (BMG LABTECH, Ortenberg, Germany). Fluorescence (λex = 320 nm, λem = 420 nm) was measured every min over an incubation period of 60 min. All measurements were conducted with three replicates of each variant consisting of IQFS in buffer solution (variant 1), IQFS in buffer solution with *S. griseus* protease solution (variant 2), IQFS in buffer-pectin solution (variant 3), IQFS in buffer-pectin-solution with *S. griseus* protease solution (variant 4).

### 2.4. Absorbance Assay

The main measurement was conducted in a flat-bottom 96-well plate using 3,5-dinitro salicylic acid (3,5 DNS) as a colorimetric agent as described previously [18,19] with slight modifications. Briefly, the carbohydrase substrate (cellulose/ pectin/ starch) was dissolved in the borate-phosphate buffer. Then, 10 µL of enzyme preparation as provided by the manufacturer (α-amylase or Viscozym^®^ L) was added to 1 mL of substrate suspension in safe-lock Eppendorf tubes. Immediately afterwards, a freshly prepared *S. griseus* protease solution (0.58 U/mL) was added. For the preliminary experiments, 5 (dose 1), 25 (dose 2) and 50 µL (dose 3) of *S. griseus* protease solution were added to the carbohydrase-substrate mixture. For the main experiments, only 25 µL of *S. griseus* protease solution were used. Reaction mixtures were incubated at 39 °C at 900 rpm in an Eppendorf Thermomixer C (Eppendorf, Hamburg, Germany). Incubation times of 0, 3, and 5 h were used in the preliminary experiments and 0, 1, 2, 3 and 5 h in the main ones. The incubation times were based on the conditions set by the *S. griseus* protease test [1,3]. At these time points, 12 µL of the reaction mixture was mixed with 24 µL 3,5-DNS. The mixture was heated at 95 °C for 5 min in HBT 130-2 block thermostat (Haep Labor Consult Bio Tech, Bovenden, Germany) and cooled down in an ice bath for up to 1 min. Then, 160 µL bi-distilled water were added and subsequently centrifuged for 10 s at 11,000× *g*. A total of 180 µL of the final mixture were pipetted into a 96 flat-bottom microwell plate (Greiner Bio-One GmbH, Frickenhausen, Germany). The 3-nitrogroup of 3,5-DNS is reduced by the aldehyde function of the monomeric sugars forming 3-amino, 5-nitrosalicylic acid resulting in an intensive color change. The absorbance was measured at λ = 540 nm using a Tecan Sunrise absorbance microplate reader (Tecan Trading AG, Männedorf, Switzerland). The absorbance values were corrected by blanks containing specific enzyme and substrate-buffer solution. In order to convert absorbance units into product concentrations, calibration curves using glucose in the respective carbohydrate-buffer solutions were created. Glucose was dissolved in duplicates at 0, 0.25, 0.5, 1, and 2 mg/mL in borate-phosphate buffer (pH 6.75) containing cellulose, pectin, and starch solution at 20 mg/mL *(w/v*) [18]. All measurements were conducted with three replicates of each variant consisting of carbohydrase-substrate with Viscozym^®^ L or α-amylase (variant 1) and carbohydrase-substrate with Viscozym^®^ L or α-amylase and, in each case, *S. griseus* protease solution (variant 2).

### 2.5. Statistical Analysis

Statistical analysis was performed using SAS 9.4 (SAS Institute Inc., Cary, NC, USA). Least squares means of fluorescence units (FU) and reducing sugar concentrations were estimated using the MIXED procedure and the model given below, separately for each variant and each incubation time. The Gaussian distribution of studentized residuals was confirmed using the UNIVARIATE procedure. For the analysis of FU and reducing sugar concentrations in the dose–response relationship, repeated measures and residual effects were considered specific for treatment *j*. For the analysis of reducing sugar concentrations of the main experiment, homogenous residual variances were considered. 

The used mixed model was:

*Y_ijk_* = *µ* + *α_i_* + *β_j_* + (*αβ*)*_ij_* + *r_jk_* + *e_ijk_*,

where *Y_ijk_* is FU, or reducing sugar concentration (mg/mL),

*µ* is the general mean, 

*α_i_* is the fixed effect of time level *i* (*i* = 0, …, 60 min in the fluorescence experiment, *i* = 0, 3, 5 h in the dose–response experiment and *i* = 0, …,5 h in the main experiment), 

*β_j_* is the fixed effect of variant *j* (*j* = 1, …, 4 in the fluorescence experiment, *j* = 1, …, 4, where 1 = 0 µL of *S. griseus* protease solution, 2 = dose 1, 3 = dose 2, 4 = dose 3 in the dose–response experiment and *j* = 1, 2 in the main experiment), 

(*αβ*)*_ij_* is the interaction of level *i* for α and level *j* for *β*, 

*r_jk_* is the random effect of repetition *k* (1, …, 3) within level *j* of treatment *β* with *r_jk_*~N (0, σ^2^*r_j_*) and

*e_ijk_* is the random residual effect with *e_ijk_*~N (0, σ^2^*e_j_*). Differences among least squares means with *p* < 0.05 were considered to be significant.

## 3. Results

Calibration curves using glucose in the respective carbohydrate-buffer solutions (0, 0.25, 0.5, 1.0, and 2.0 mg/mL) yielded regression slopes with a coefficient of determination of R^2^ = 0.973, R^2^ = 0.993 and R^2^ = 0.975 in cellulose, pectin and starch solution, respectively.

### 3.1. Preliminary Experiments

Incubation of IQFS peptides with *S. griseus* protease solution increased fluorescent units immediately after the start of the incubation (*p* < 0.05). The pectin in the buffer-substrate solution itself significantly increased fluorescence units after 3 and 6 min in IQFS 1 and IQFS 2, respectively (*p* < 0.05) (Table 1).

**Figure 1:**
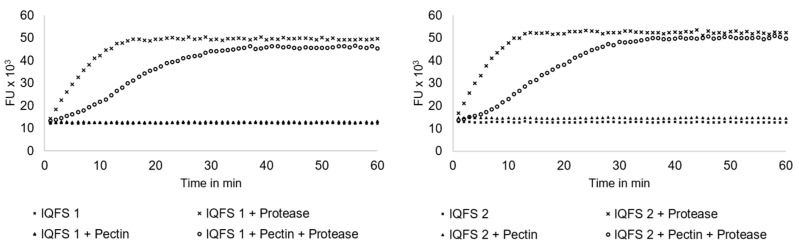
Means of fluorescent units from internally quenched fluorogenic substrate hydrolyzed by *Streptomyces griseus* protease mixture with and without pectin during 60 min of incubation. IQFS: internally quenched fluorogenic substrate of the general structure: 2-amino benzoic acid-alanine-alanine-Xaa-phenylalanine-alanine-alanine-lysine-2,4-dinitrophenol, where IQFS 1: Xaa = alanine; IQFS 2: Xaa = arginine. All values are given in fluorescent units (FU). Fluorescence was measured in a fluorescence plate reader (λex = 320 nm, λem = 420 nm). Pectin was dissolved at 20 mg/mL, *S. griseus* protease mixture at 0.58 U/mL and heptapeptides at 100 µM in borate-phosphate buffer at pH 6.75. A total of 2.5 µL of *S. griseus* protease solution was used.

**Figure 2:**
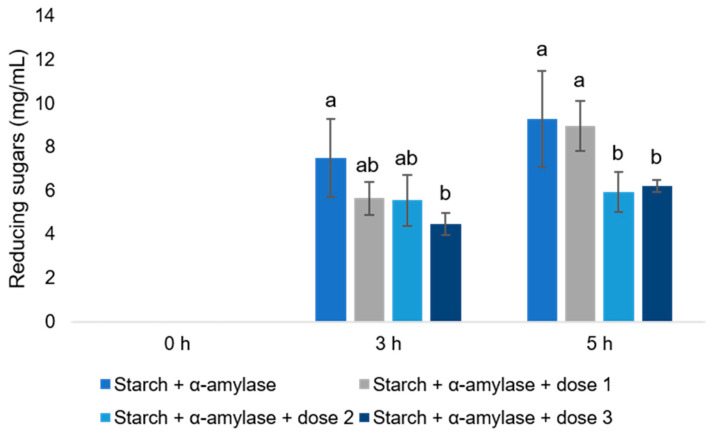
Means with standard deviation of reducing sugars from starch degraded by α-amylase with increased doses of *Streptomyces griseus* protease solution. a, b: different letters indicate significant differences within incubation time point between *S. griseus* protease doses (*p* < 0.05); dose 1: 5 µL protease solution; dose 2: 25 µL protease solution; dose 3: 50 µL protease solution Starch was dissolved at 20 mg/mL and *S. griseus* protease mixture at 0.58 U/mL in borate-phosphate-buffer at pH 6.75. Error bars show the standard deviation.

Over the entire incubation period, maximum fluorescence was reached after 16 min for IQFS 1 and 13 min for IQFS 2 (Figure 1). For the pectin-containing buffer, the maximum fluorescence was detected after 36 min for IQFS 1 and after 40 min for IQFS 2 (Figure 2).

The increasing dose of *S. griseus* protease solution (5, 25 and 50 µL) resulted in different responses regarding the time-dependent release of reducing sugars from starch degraded by α-amylase (control) (Figure 2 and Table S1). The 5 µL protease solution dose had no significant effect on reducing sugar concentrations at incubation times of 3 h and 5 h, respectively (*p* > 0.05). At a dose of 25 µL of protease solution, a significant decrease in reducing sugar concentrations by 37% was observed at an incubation time of 5 h (*p* < 0.05). The dose of 50 µL protease solution led to a significant decrease in reducing sugar concentrations at 3 h (40%) and 5 h (33%), respectively, compared to the control (*p* < 0.05) (Figure 1, Table S1). 

Based on these results, the dose of 25 µL protease solution seems to be appropriate to investigate any impact of *S. griseus* protease on the release of reducing sugars by α-amylase and Viscozym^®^ L from complex carbohydrates during a 5 h co-incubation. It avoids over- or under-dosing of the *S. griseus* protease and reduces the release of reducing sugars by α-amylase by a similar amount compared to the 50 µL protease dose.

### 3.2. Main Experiments

The effect of co-incubated *S. griseus* protease and carbohydrase (α-amylase/Viscozym^®^ L) on the release of reducing sugars was investigated using cellulose, pectin and starch as substrates during a 5 h co-incubation (Table 2, Table 3, Table 4 and Table 5; Figure S1). The coefficient of determination of carbohydrate degradation by carbohydrase at different incubation times is shown in Table S2. The coefficient serves as a parameter for the evaluation of the results, as an evaluation can only be carried out in the linear range between the incubation times [20].

The co-incubation of Viscozym^®^ L and *S. griseus* protease resulted in a significant increase in reducing sugar concentrations by 15% with an incubation time of 3 h and with pectin as substrate (*p* < 0.05) (Table 2 and Figure S1). 

The co-incubation of α-amylase and protease resulted in a decrease in reducing sugar concentrations by 3% at 5 h incubation time with starch as substrate (Table 3 and Figure S1).

The co-incubation of Viscozym^®^ L resulted in a decrease in reducing sugar concentrations by 37% with 5 h of incubation time and starch as substrate (Table 4 and Figure S1).

The differences resulting from cellulose degradation between Viscozym^®^ L and co-incubation of both enzymes were low (*p* > 0.05) (Table 5 and Figure S1). 

## 4. Discussion

The aim of the study was to investigate the effects of the co-incubation of the *S. griseus* protease and carbohydrase on the release of reducing sugars from different carbohydrates under the conditions set by the *S. griseus* protease test at selected incubation times [1]. 

The coefficient of determination of linear regression was added to give information on whether degradation occurred within the linear range. Results outside of the linear range should be carefully interpreted [20]. 

We decided to use the commercial enzyme preparations α-amylase (synonym: Termamyl^®^ 2X) and Viscozym^®^ L, as they had already been used as pre-incubation agents in a study in which ruminal protein degradation was determined using *S. griseus* protease [7]. Viscozym^®^ L appears to be advantageous as a co-incubation agent in the *S. griseus* protease test, as the enzyme mixture provides multiple cell wall-degrading enzymes for extensive hydrolysis of complexes of protein and fiber/starch. 

To our knowledge, the exact enzyme composition of Viscozym^®^ L is actually not known. Uncontrollable interactions within the *S. griseus* protease and Viscozym^®^ L mixtures or between protease and α-amylase or Viscozym^®^ L could have contributed to high standard deviations. A certain inhomogeneity of the substrate-buffer solution, an emulsion of cellulose and starch, was probably another source of bias. Additionally, sucrose was added to the carbohydrase preparations according to the manufacturer’s information. As a non-reducing substance, sucrose could interact with 3,5-DNS during incubation by enzymatic degradation or dissolution in buffer, resulting in increased absorbance units. 

According to the manufacturer, the *S. griseus* protease mixture has its highest activity at pH 8.8, α-amylase between pH 6–7/>80 °C, and Viscozym^®^ L at pH 3.5–5.5/ 55 °C [12]. The incubation conditions set for the standardized *S. griseus* protease test (39 °C and pH 6.75) do not correspond to the optimal conditions of either the α-amylase, Viscozym^®^ L or the *S. griseus* protease mixture. However, the release of reducing sugars by carbohydrases was determined for different carbohydrates.

The *S. griseus* protease was proved to be active immediately after adding to IQFS in borate-phosphate buffer at pH 6.75. A lag time in increasing fluorescence units was observed with pectin as an additional supplement in the buffer solution (Table 1, Figure 1). Pectin is a viscous component that might have influenced *S. griseus* protease by decelerating the degradation of the peptides (IQFS). It can be assumed that pectin forms a gel-like emulsion with peptides [21] and this resulted in decelerating degradation through *S. griseus* protease.

The relationship between increasing *S. griseus* protease mixture doses and the release of reducing sugars by α-amylase showed that the reducing sugar concentrations declined in relation to an increasing dosage of protease solution. The results revealed that the *S. griseus* protease dose of 25 µL (0.58 U/mL) appears to be useful for further experiments in this study.

The final results regarding the co-incubation of carbohydrases (α-amylase/Viscozym^®^ L) and *S. griseus* protease revealed that reducing sugar concentrations decreased up to 37% after 5 h of incubation compared to an incubation without protease using starch as substrate. To give a practical example, literature data showed an apparent influence of *Aspergillus saitoi* protease in co-incubation with cellulase, pectinase, xylanase or glucanase as dry matter and crude protein digestibility of maize and a soybean-maize mixture decreased. They suspect that the protease degrades the carbohydrases [10,11]. However, it may be difficult to determine carbohydrase level after co-incubation because of the secondary effects of the enzyme protein during incubation. These are the denaturation and sticking of the enzyme protein to the wall of the reaction vessel, which would lead to incorrect determination of the carbohydrase level [20].

The co-incubation of Viscozym^®^ L and *S. griseus* protease with pectin and cellulose as substrates showed no significant influence. 

However, it is difficult to interpret the results, knowing that Viscozym^®^ L and *S. griseus* protease preparation are enzyme mixtures with unknown enzyme compositions, which makes it quite difficult to determine the enzyme activity. Each enzyme in these mixtures has its own substrate specificity and affinity. If one substrate’s release of reducing sugars was reduced in the co-incubation of carbohydrase and *S. griseus* protease, the reduction is not necessarily transferable to other substrates. It remains uncertain whether the activity of each enzyme in the enzyme mixture is equally reduced. In the case of *S. griseus* protease mixture, it remains unclear how high the affinity of each single protease to the carbohydrase as a potential substrate is. It remains unclear whether a single enzyme or all enzymes cause the observed influence of the *S. griseus* protease mixture on the carbohydrase. Based on these results, influence could be observed, which presumably becomes more effective at an incubation time of 5 h. An additional reason for the moderate influence of *S. griseus* protease could be related to autolysis. According to the manufacturer, the *S. griseus* protease mixture contains a serine protease known for autolysis loop [22]. Over the incubation period of 5 h, the hydrolysis might inactivate some of the proteases within the mixture, resulting in lower proteolytic potential.

## 5. Conclusions

Despite predominantly not being significant, the co-incubation of α-amylase or a cellulolytic enzyme mixture with *Streptomyces griseus* protease decreased the release of reducing sugars from different carbohydrates. From a methodical point of view, co-incubation of protease and carbohydrase during the *Streptomyces griseus* protease test still appears promising, and further studies should be intended to investigate which carbohydrase activities are required to counteract the disturbing protease effect on carbohydrases. 

## Figures and Tables

**Table 1:** Means of fluorescent units from internally quenched fluorogenic substrate hydrolyzed by *Streptomyces griseus* protease mixture with and without pectin measured for the first 10 min of incubation.

	IQFS 1 +		*p*-Value	IQFS 1 +		*p*-Value	IQFS 2 +		*p*-Value	IQFS 2 +		*p*-Value
Time (min)	Buffer	Buffer + Protease	Time × Variant	Pectin	Pectin + Protease	Time × Variant	Buffer	Buffer + Protease	Time × Variant	Pectin	Pectin + Protease	Time × Variant
0	12.2 ^b^	14.3 ^a^	<0.0001	12.6 ^a^	13.3 ^a^	0.4883	12.9 ^b^	16.7 ^a^	0.0005	14.7 ^a^	13.6 ^a^	0.3769
1	12.2 ^b^	18.4 ^a^	<0.0001	12.6 ^a^	13.8 ^a^	0.2105	12.9 ^b^	21.1 ^a^	<0.0001	14.8 ^a^	14.0 ^a^	0.5406
2	12.2 ^b^	22.4 ^a^	<0.0001	12.8 ^a^	14.6 ^a^	0.0689	12.8 ^b^	25.8 ^a^	<0.0001	15.1 ^a^	15.1 ^a^	0.9771
3	12.2 ^b^	26.0 ^a^	<0.0001	12.7 ^b^	15.5 ^a^	0.0040	12.8 ^b^	30.0 ^a^	<0.0001	14.9 ^a^	15.6 ^a^	0.5933
4	12.1 ^b^	29.5 ^a^	<0.0001	12.5 ^b^	16.2 ^a^	0.0001	12.8 ^b^	33.4 ^a^	<0.0001	14.7 ^a^	16.3 ^a^	0.2260
5	12.1 ^b^	32.6 ^a^	<0.0001	12.7 ^b^	17.2 ^a^	<0.0001	12.7 ^b^	37.6 ^a^	<0.0001	14.7 ^a^	17.3 ^a^	0.0640
6	12.1 ^b^	35.8 ^a^	<0.0001	12.8 ^b^	17.9 ^a^	<0.0001	12.8 ^b^	41.0 ^a^	<0.0001	14.8 ^b^	18.4 ^a^	0.0096
7	12.2 ^b^	38.1 ^a^	<0.0001	12.6 ^b^	19.2 ^a^	<0.0001	12.8 ^b^	43.4 ^a^	<0.0001	14.6 ^b^	19.7 ^a^	0.0003
8	12.2 ^b^	40.9 ^a^	<0.0001	12.5 ^b^	20.4 ^a^	<0.0001	12.8 ^b^	45.5 ^a^	<0.0001	14.6 ^b^	21.5 ^a^	<0.0001
9	12.3 ^b^	42.2 ^a^	<0.0001	12.6 ^b^	21.6 ^a^	<0.0001	12.8 ^b^	47.8 ^a^	<0.0001	14.7 ^b^	23.0 ^a^	<0.0001
10	12.2 ^b^	44.7 ^a^	<0.0001	12.5 ^b^	22.8 ^a^	<0.0001	12.7 ^b^	49.9 ^a^	<0.0001	14.6 ^b^	25.1 ^a^	<0.0001
SD	0.1–0.2	0.0–1.2	-	0.2–0.5	0.5–2.3	-	0.8–1.2	0.5–1.5	-	0.1–0.3	1.1–2.8	-

^a, b^ different lowercase superscripts indicate significant differences within heptapeptide between buffer/protease or pectin/protease variants (*p* < 0.05); IQFS: internally quenched fluorogenic substrate of the general structure: 2-amino benzoic acid-alanine-alanine-Xaa-phenylalanine-alanine-alanine-lysine-2,4-dinitrophenol, where IQFS 1: Xaa = alanine; IQFS 2: Xaa = arginine; SD: standard deviation. All values are given fluorescent units ×10^3^. Fluorescence was measured in a fluorescence plate reader (λex = 320 nm, λem = 420 nm). Pectin was dissolved at 20 mg/mL, *S. griseus* protease mixture at 0.58 U/mL, and heptapeptides at 100 µM in borate-phosphate buffer at pH 6.75. A total of 2.5 µL of *S. griseus* protease solution was used.

**Table 2:** Means of reducing sugar concentration (mg/mL) from pectin degraded by Viscozym^®^ L in presence of *Streptomyces griseus* protease mixture during 5 h of incubation.

	Reducing Sugar Concentration			*p*-Value
Time (h)	Pectin	Pectin + Viscozym L	Pectin + Viscozym L + Protease	Time × Variant
0	0.3	0 ^a^	0 ^a^	10.000
1	0	2.6 ^a^	2.5 ^a^	0.9889
2	0	4.6 ^a^	4.1 ^a^	0.1636
3	0	4.7 ^b^	5.4 ^a^	0.0304
5	0	5.9 ^a^	5.8 ^a^	0.6590
Range of SD	0–0.1	0–0.5	0–0.7	-

^a, b^ different lowercase superscripts indicate significant differences for an incubation time point (*p* < 0.05); SD: standard deviation. Pectin was dissolved at 20 mg/mL and *S. griseus* protease mixture at 0.58 U/mL in borate-phosphate buffer at pH 6.75. A total of 10 µL of Viscozym^®^ L as provided by the manufacturer and 25 µL of *S. griseus* protease solution was used in the experiment.

**Table 3:** Means of reducing sugar concentration (mg/mL) from starch degraded by α-amylase in presence of *Streptomyces griseus* protease mixture during 5 h of incubation.

	Reducing Sugar Concentration			*p*-Value
Time (h)	Starch	Starch + α-Amylase	Starch + α-Amylase + Protease	Time × Variant
0	0.1	0 ^a^	0 ^a^	10.000
1	0.1	4.2 ^a^	4.0 ^a^	0.6507
2	0.1	6.2 ^a^	5.9 ^a^	0.4366
3	0.1	7.0 ^a^	7.1 ^a^	0.9124
5	0.1	9.2 ^a^	8.9 ^a^	0.5111
Range of SD	0	0–0.6	0–1.3	-

^a, b^ different lowercase superscripts indicate significant differences for an incubation time point (*p* < 0.05); SD: standard deviation. Starch was dissolved at 20 mg/mL and *S. griseus* protease mixture at 0.58 U/mL in borate-phosphate buffer at pH 6.75. A total of 10 µL of α-amylase as provided by the manufacturer and 25 µL of *S. griseus* protease solution was used in the experiment.

**Table 4:** Means of reducing sugar concentration (mg/mL) from starch degraded by Viscozym^®^ L in presence of *Streptomyces griseus* protease mixture during 5 h of incubation.

	Reducing Sugar Concentration			*p*-Value
Time (h)	Starch	Starch + Viscozym L	Starch + Viscozym L + Protease	Time × Variant
0	0.2	0 ^a^	0 ^a^	10.000
1	0.1	0.9 ^a^	0.3 ^a^	0.3150
2	0.2	1.2 ^a^	0.9 ^a^	0.5643
3	0.1	1.3 ^a^	1.1 ^a^	0.6843
5	0.1	2.7 ^a^	1.7 ^a^	0.0680
Range of SD	0	0–1.1	0–0.9	-

^a, b^ different lowercase superscripts indicate significant differences for an incubation time point (*p* < 0.05); SD: standard deviation. Starch was dissolved at 20 mg/mL and *S. griseus* protease mixture at 0.58 U/mL in borate-phosphate buffer at pH 6.75. A total of 10 µL of Viscozym^®^ L as provided by the manufacturer and 25 µL of *S. griseus* protease solution was used in the experiment.

**Table 5:** Means of reducing sugar concentration (mg/mL) from cellulose degraded by Viscozym^®^ L in presence of *Streptomyces griseus* protease mixture during 5 h of incubation.

	Reducing Sugar Concentration			*p*-Value
Time (h)	Cellulose	Cellulose + Viscozym L	Cellulose + Viscozym L + Protease	Time × Variant
0	0	0.1 ^a^	0.6 ^a^	0.3379
1	0.1	0.8 ^a^	0.5 ^a^	0.5868
2	0	0.9 ^a^	0.8 ^a^	0.9552
3	0.1	1.3 ^a^	1.2 ^a^	0.7714
5	0	1.7 ^a^	1.6 ^a^	0.9009
Range of SD	0–0.1	0.1–1.2	0.4–1.1	-

^a, b^ different lowercase superscripts indicate significant differences for an incubation time point (*p* < 0.05); SD: standard deviation. Cellulose was dissolved at 20 mg/mL and *S. griseus* protease mixture at 0.58 U/mL in borate-phosphate buffer at pH 6.75. A total of 10 µL of Viscozym^®^ L as provided by the manufacturer and 25 µL of *S. griseus* protease solution were used in the experiment.

## Data Availability

The data presented in this study are available on request from the corresponding author.

## Supplementary Materials

The following supporting information can be downloaded at: https://www.mdpi.com/article/10.3390/ani14131931/s1, Table S1 Means with standard deviation (SD) of reducing sugars from starch degraded by α-amylase with increased dosage of *Streptomyces griseus* protease solution; Table S2: Coefficient of determination (R^2^) of substrates degraded by α-amylase or Viscozym^®^ L in presence of *Streptomyces griseus* protease mixture during 5 h of incubation; Figure S1: Means of reducing sugar concentrations from substrates degraded by Viscozym^®^ L or α-amylase in presence of *Streptomyces griseus* protease during 5 h of incubation.
