# Supplementary material for: The Impact of Streptomyces griseus Protease Reserved for Protein Evaluation of Ruminant Feed on Carbohydrase Activity during Co-Incubation
Source: Animals (Basel). 2024 Jun 29;14(13):1931. doi: 10.3390/ani14131931 (PMC11240829; doi:10.3390/ani14131931)
Supplement: Supplementary file 1 [file animals-14-01931-s001.zip › animals-3021574-supplementary.pdf]

Supplements

Table S1: Means with standard deviation (SD) of reducing sugar concentration from starch degraded by α-amylase with increased dosage of *Streptomyces griseus* protease solution

	Reducing sugar concentration			
	Starch + α-amylase	Starch + α-amylase + Dose 1	Starch + α-amylase + Dose 2	Starch + α-amylase + Dose 3
0	0	0	0	0
3	7.5^{a}	5.7^{ab}	5.6^{ab}	4.5^{b}
5	9.3^{a}	9.0^{a}	5.9^{b}	6.2^{b}
SD	0 – 2.2	0 – 1.1	0 – 1.2	0 – 0.5

$^{a, b}$ different letters indicate significant differences within incubation time point between protease doses ($p < 0.05$); dose 1: 5 µL protease solution; dose 2: 25 µL protease solution; dose 3: 50 µL protease solution

Starch was dissolved at 20 mg/mL and *S. griseus* protease mixture at 0.58 U/mL in borate-phosphate-buffer at pH 6.75.

Table S2: Coefficient of determination (R^2) of substrates degraded by α-amylase or Viscozym® L in presence of *Streptomyces griseus* protease mixture during 5 h of incubation

Time (h)	R^2 of reducing sugar concentration							
	Pectin + Viscozym L	Pectin + Viscozym L + Protease	Starch + α-amylase	Starch + α-amylase + Protease	Starch + Viscozym L	Starch + Viscozym L + Protease	Cellulose + Viscozym L	Cellulose + Viscozym L + Protease
0	-	-	-	-	-	-	-	-
1	1	1	1	1	1	1	1	1
2	0.996	0.981	0.962	0.960	0.932	0.978	0.794	0.371
3	0.892	0.976	0.903	0.929	0.843	0.967	0.903	0.748
5	0.838	0.835	0.882	0.886	0.946	0.978	0.912	0.913

The coefficient of determination of the linear regression was determined from 0 h incubation time to the specific incubation time by including preceding timepoints.

Pectin, starch and cellulose was dissolved at 20 mg/mL, respectively and *S. griseus* protease mixture at 0.58 U/mL in borate-phosphate buffer at pH 6.75.

10 µL of Viscozym® L or α-amylase as provided by the manufacturer, respectively and 25 µL of *S. griseus* protease solution was used in the experiment.

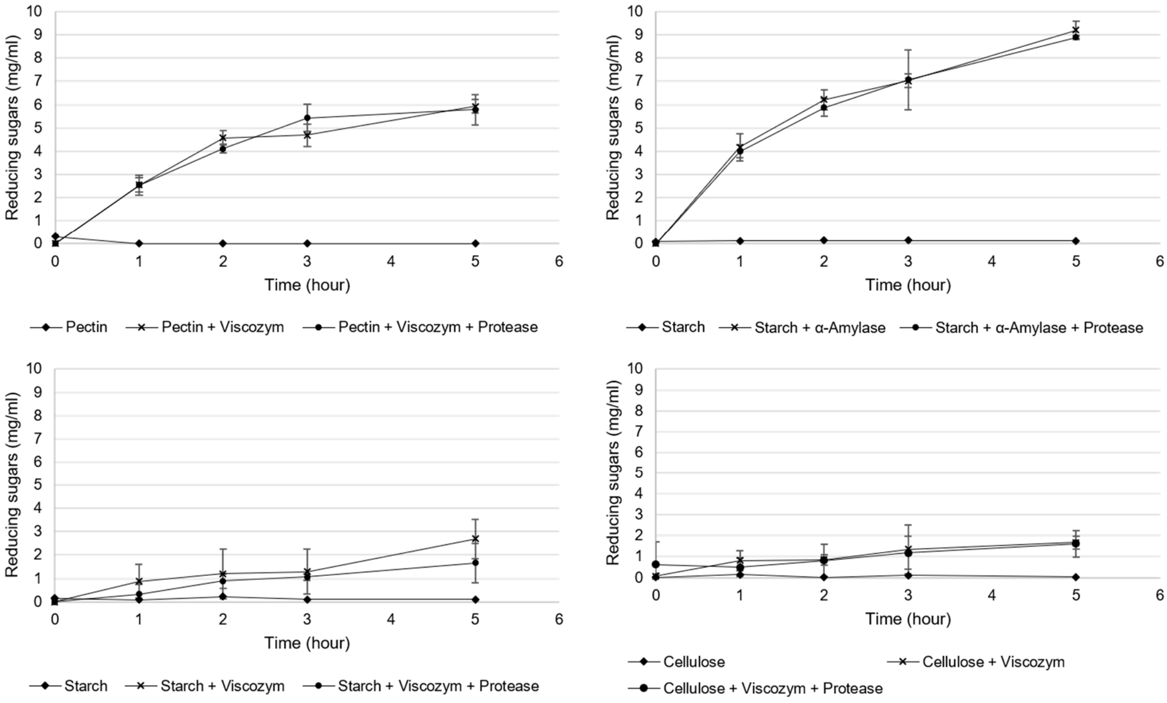

Pectin, starch and cellulose was dissolved at 20 mg/mL, respectively, and *Streptomyces griseus* protease at 0.58 U/mL in borate-phosphate buffer at pH 6.75.

10 µL of Viscozym® L or α-amylase as provided by manufacturer, respectively and 25 µL of *S. griseus* protease solution was used in experiment.

Figure S1: Means of reducing sugar concentrations from substrates degraded by Viscozym® L or α-amylase in presence of *Streptomyces griseus* protease during 5 h of incubation
